# Intra‐Arterial Thrombolysis After Successful Thrombectomy: A Systematic Review and Meta‐Analysis of Randomized Controlled Trials

**DOI:** 10.1161/SVIN.125.001847

**Published:** 2025-07-31

**Authors:** Tianqi Xu, Chushuang Chen, Vignan Yogendrakumar, Dennis J Cordato, Christopher Blair, Timmy Pham, Andrew K Cheung, Nathan W Manning, Mark W Parsons, Longting Lin

**Affiliations:** ^1^ South Western Sydney Clinical School UNSW Liverpool New South Wales Australia; ^2^ Ingham Institute for Applied Medical Research Liverpool New South Wales Australia; ^3^ Division of Neurology Department of Medicine The Ottawa Hospital Ottawa Hospital Research Institute University of Ottawa Ottawa Ontario Canada; ^4^ Department of Neurology Liverpool Hospital Sydney New South Wales Australia; ^5^ Department of Neurointerventional Radiology Liverpool Hospital Sydney New South Wales Australia

**Keywords:** acute ischemic stroke, endovascular thrombectomy, expanded Thrombolysis in Cerebral Infarction, intra‐arterial thrombolysis, randomized controlled trials

## Abstract

**Background:**

This study aims to conduct a systematic review and meta‐analysis of randomized controlled trials (RCTs) to assess the efficacy and safety of intra‐arterial thrombolysis (IAT) following successful endovascular thrombectomy (EVT) in patients with stroke.

**Methods:**

A systematic literature search was conducted to identify RCTs comparing IAT versus no IAT after successful EVT. The primary efficacy outcome was a modified Rankin Scale score of 0–1 at 90 days, and the primary safety outcomes included symptomatic intracranial hemorrhage and 90‐day mortality. Subgroup meta‐analyses were conducted based on expanded Thrombolysis in Cerebral Infarction (eTICI) and prior intravenous thrombolysis (IVT). Both random‐effects and common‐effect models were applied with model selection determined by the level of heterogeneity.

**Results:**

Six RCTs were included, comprising 990 patients in the IAT group and 981 in the control group. Meta‐analysis demonstrated that IAT following successful EVT improved the rate of disability‐free survival at 90 days, with a pooled risk ratio (RR) of 1.24 (95% CI: 1.12–1.39) and no substantial heterogeneity (I^2^ = 16.0%, *P* = 0.31). Additionally, IAT treatment did not increase the risk of symptomatic intracranial hemorrhage (RR: 1.14 [95% CI: 0.85–1.54]) or 90‐day mortality (RR: 1.05 [95% CI: 0.87–1.26]). Subgroup meta‐analysis suggested greater benefits from IAT in patients with eTICI 2b50/67 (RR: 1.51 [95% CI: 1.03–2.23]) than in those with eTICI 2c/3 (RR: 1.22, 95% CI: 0.99–1.50), and in patients without prior IVT (RR: 1.33 [95% CI: 1.08–1.65]) compared with those who received IVT (RR: 1.17 [95% CI: 0.85–1.62]).

**Conclusion:**

IAT following successful EVT improved 90‐day functional outcomes without increasing the risk of symptomatic intracranial hemorrhage or 90‐day mortality. Patients in the eTICI 2b50/67 subgroup and those without prior IVT showed a trend toward greater benefit from IAT compared with the eTICI 2c/3 subgroup and those who received IVT prior to thrombectomy.

**Nonstandard Abbreviations and Acronyms:**

eTICI: expanded Thrombolysis in Cerebral Infarction
EVT: endovascular thrombectomy
IAT: intra‐arterial thrombolysis
IVT: intravenous thrombolysis
sICH: symptomatic intracranial hemorrhage

**Clinical Perspective**

- This study indicated that intra‐arterial thrombolysis following successful endovascular thrombectomy in patients with stroke is safe without increasing the risk of symptomatic intracranial hemorrhage and 90‐day mortality.
- Intra‐arterial thrombolysis following successful endovascular thrombectomy significantly improved the rate of disability‐free survival at 90 days, particularly in patients without complete recanalization (expanded Thrombolysis in Cerebral Infarction 2b50/67) and those without prior intravenous thrombolysis.
- The therapeutic effect of intra‐arterial thrombolysis may be further improved if patient selection in future studies is refined by identifying with microcirculation dysfunction after successful endovascular thrombectomy. This might be achieved using digital subtraction angiography “perfusion” and flat‐panel computed tomography perfusion.

Endovascular thrombectomy (EVT) is the preferred treatment for patients with acute ischemic stroke with large vessel occlusion.^1^ However, despite 71% of patients achieving successful large vessel recanalization (modified Thrombolysis in Cerebral Infarction 2b‐3) following timely EVT, only 46.0% achieved good clinical outcomes (modified Rankin Scale [mRS] score 0–2) at 90 days.^2^ Several randomized controlled trials (RCTs) have evaluated whether intra‐arterial thrombolysis (IAT) after successful EVT improves clinical outcomes. However, findings between trials remain inconsistent. The POST‐UK (Adjunctive Intra‐Arterial Urokinase After Near‐Complete to Complete Reperfusion for Acute Ischemic Stroke),^3^ POST‐TNK (Adjunctive Intra‐Arterial Tenecteplase Following Near‐Complete to Complete Reperfusion for Large Vessel Occlusion Stroke),^4^ and ATTENTION‐IA (Intra‐Arterial Tenecteplase After Successful Endovascular Recanalisation in Patients With Acute Posterior Circulation Arterial Occlusion)^5^ trials found that IAT did not significantly improve functional outcomes. In contrast, the CHOICE (Intraarterial Alteplase Versus Placebo After Mechanical Thrombectomy) trial^6^ and the latest results from the ANGEL‐TNK (Intra‐Arterial Recombinant Human TNK Tissue‐Type Plasminogen Activator Thrombolysis for Acute Large Vascular Occlusion After Successful Mechanical Thrombectomy Recanalization)^7^ and PEARL (Intra‐Arterial Alteplase for Acute Ischaemic Stroke After Mechanical Thrombectomy)^8^ trials suggest that IAT following successful EVT significantly improves the rate of disability‐free survival at 90 days.

To address these discrepancies, we conducted a systematic review and meta‐analysis of RCTs to comprehensively assess the efficacy and safety of IAT following successful EVT in patients with acute ischemic stroke.

## Methods

Data are available from the corresponding author upon reasonable request.

### Search Strategy and Selection Criteria

Following the Preferred Reporting Items for Systematic Reviews and Meta‐Analyses guidelines,^9^ we conducted a systematic literature search in PubMed and EMBASE databases up to March 3, 2025. Additionally, we included findings presented at the International Stroke Conference in Los Angeles on February 7, 2025.^7^, ^8^ Our study protocol was registered in the International Prospective Register of Systematic Reviews under registration number CRD420251007052.

The study selection process was independently reviewed by both T.X. and C.C., including duplicate removal, title and abstract screening, and full‐text review. Any discrepancies between the 2 reviewers were resolved through further discussion to reach a consensus. Additionally, citation tracking of relevant references was performed to identify any additional eligible studies. The detailed search strategy, including specific search terms, Boolean operators (AND/OR), Medical Subject Headings, and field restrictions (eg, title and abstract searches), was provided in the Supplementary Material.

We identified RCTs that met the following criteria: (1) studies comparing IAT versus no IAT following successful EVT in patients with acute ischemic stroke; (2) successful EVT was defined as expanded TICI (eTICI) score of 2b‐3;^10^ (3) studies reporting both the primary efficacy outcome (90‐day mRS score) and the primary safety outcomes (symptomatic intracranial hemorrhage [sICH] and 90‐day mortality). Exclusion criteria included non‐RCT studies, post hoc analyses of RCTs, review articles, meta‐analyses, and studies investigating IAT administration either before EVT or in patients with unsuccessful EVT. For studies that published only RCT protocols, we conducted an additional Internet search to determine whether further results had been presented at conferences or published in other formats.

### Quality Assessment

We assessed the quality of the included RCTs using the Risk of Bias 2.0 tool,^11^ which evaluated 5 key domains: randomization process, deviations from the intended interventions, missing outcome data, measurement of the outcome, and selection of the reported result.

### Data Extraction

The extracted data included RCT protocol details, such as study design, sample size, inclusion and exclusion criteria, intervention and control specifications, as well as the dosage and type of thrombolytic agent used for IAT. Additionally, study characteristics were collected, including age, sex, baseline National Institutes of Health Stroke Scale score, baseline Alberta Stroke Program Early CT [Computed Tomography] Score, prior intravenous thrombolysis (IVT), occlusion site, eTICI score, and time from stroke onset to randomization. Furthermore, primary outcome measures were documented, including 90‐day mRS score, sICH, and 90‐day mortality.

### Data Synthesis and Analysis

Statistical analyses were performed using R version 4.4.2 (R Foundation, Vienna, Austria). Both the random‐effects model and common‐effect model were applied to calculate pooled risk ratio (RR), with model selection based on the level of heterogeneity. According to the Cochrane Handbook,^11^ heterogeneity was classified as follows: I^2^ = 0%–40%: may not be important; I^2^ = 30%–60%: may indicate moderate heterogeneity; I^2^ = 50%–90%: may indicate substantial heterogeneity; I^2^ = 75%–100%: represents considerable heterogeneity. In this study, a random‐effects model was used when I^2^ exceeded 30%, indicating at least moderate heterogeneity; a common‐effect model was applied otherwise.^12^, ^13^ Leave‐one‐out analysis was conducted as a sensitivity analysis to assess the robustness of the results. In subgroup meta‐analyses, χ^2^ tests were performed to evaluate potential differences between subgroups.

## Results

### Literature Search and RCTs Characteristics

**Figure 1:**
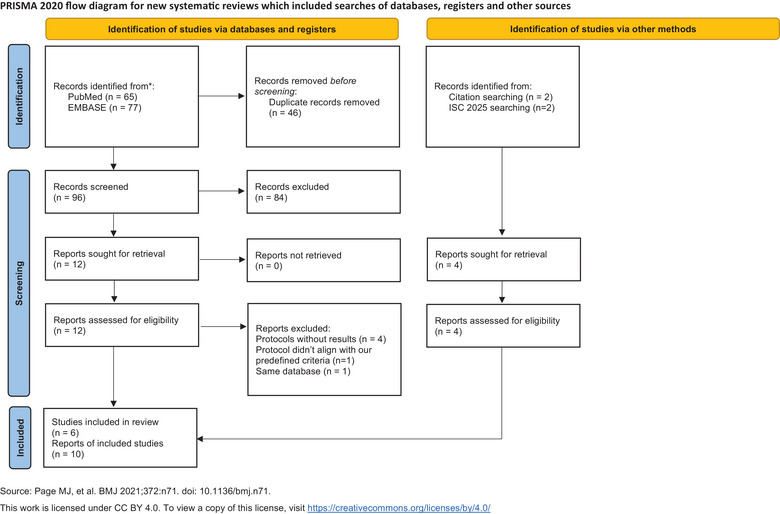
**PRISMA flow diagram illustrating the study selection process**. ISC indicates International Stroke Conference; PRISMA, Preferred Reporting Items for Systematic Reviews and Meta‐Analyses.

Figure 1 illustrates the process of literature search and screening process based on the Preferred Reporting Items for Systematic Reviews and Meta‐Analyses 2020 flow diagram.^14^ A total of 6 RCTs investigating the efficacy and safety of IAT following successful EVT were identified. Among them, 4 RCTs have been fully published with results, and the remaining 2 have published only their protocols but have presented their results at the International Stroke Conference 2025 conference. Regarding thrombolytic agents, 3 studies used intra‐arterial tenecteplase, 2 used alteplase, and 1 investigated urokinase. In terms of occlusion sites, 5 studies focused on anterior circulation occlusions, and 1 examined posterior circulation occlusions. The protocols of these 6 RCTs are summarized in Table S1.

We performed a quality assessment using the Risk of Bias 2.0 tool, and all assessed studies demonstrated a low risk of bias, indicating high methodological quality. The quality assessment of ANGEL‐TNK and PEARL was conducted based on their published protocols^15^, ^16^ and the results presented at the International Stroke Conference 2025^7^, ^8^ (Figure S1).

Table 1 summarizes the study characteristics of the 6 RCTs with reported results. The ATTENTION trial included the youngest population, with an average age of 65 years in the treatment group and 67.3 years in the control group, whereas the CHOICE study had the oldest cohort, with a median age of 73 years. Additionally, patients in the ATTENTION trial presented with the most severe admission symptoms, with a median National Institutes of Health Stroke Scale score of 19 in the treatment group and 23 in the control group, likely due to its exclusive inclusion of posterior circulation occlusion cases. POST‐UK, POST‐TNK, and ANGEL‐TNK included only patients who had not received IVT prior to EVT. The CHOICE study had the highest proportion of patients with IVT (61%). POST‐UK and POST‐TNK exclusively included patients who achieved eTICI 2c‐3, whereas the other 4 RCTs enrolled patients with eTICI 2b‐3 reperfusion.

**Table 1:** Summary of Study Characteristics

	POST‐UK		POST‐TNK		ANGEL‐TNK		PEARL		CHOICE		ATTENTION‐IA	
	Treatment (n = 267)	Control^†^ (n = 267)	Treatment (n = 269)	Control (n = 271)	Treatment (n = 126)	Control (n = 129)	Treatment (n = 164)	Control (n = 160)	Treatment (n = 61)	Control (n = 52)	Treatment (n = 104)	Control (n = 104)
Age, y, median (IQR) or mean ± SD	69 (59–77)	68 (58–76)	69 (59–76)	69 (59–76)	71.5 (60.6–79.5)	71.7 (61.7–79.0)	65.8 ± 12.8	65.1 ± 12.9	73 (71–76)	73 (69–67)	65.0 ± 11.3	67.3 ± 10.8
Male, n (%)	162 (60.7)	149 (55.8)	154 (57.2)	165 (60.9)	68 (54.0)	73 (56.6)	119 (72.6)	106 (66.2)	33 (54)	28 (54)	84 (80.8)	73 (70.2)
Baseline NIHSS score, median (IQR)	15 (11–19)	15 (10–19)	15 (11–20)	15 (10–20)	15 (12–19)	16 (12–19)	15 (11–17)	15 (11–18)	14 (8–20)	14 (10–20)	19.5 (12–35)	23 (14–35)
Baseline ASPECTS, median (IQR)	8 (7–9)	8 (7–9)	8 (7–9)	8 (7–9)	7 (6–8)	7 (6–8)	9 (7–10)	9 (7–10)	9 (9–10)	10 (8–10)	9 (8–10)	8 (8–10)
Prior IVT, n (%)	0	0	0	0	0	0	69 (42.1)	66 (41.3)	38 (62)	31 (60)	28 (26.9)	25 (24.0)
Occlusion site, n (%)												
ICA	57 (21.3)	68 (25.5)	57 (21.2)	59 (21.8)	33 (26.2)	38 (29.5)	25 (15.2)	20 (12.5)	7 (12)	4 (8)	0	0
M1	161 (60.3)	148 (55.4)	172 (63.9)	166 (61.3)	58 (46.0)	64 (49.6)	113 (68.9)	121 (75.6)	19 (31)	20 (39)	0	0
M2	49 (18.4)	51 (19.1)	40 (14.9)	46 (17.0)	35 (27.8)	27 (20.9)	26 (15.9)	19 (11.9)	33 (54)	28 (54)	0	0
VA	0	0	0	0	0	0	0	0	0	0	26 (25.0)	28 (26.9)
BA	0	0	0	0	0	0	0	0	0	0	75 (72.1)	69 (66.3)
PCA	0	0	0	0	0	0	0	0	0	0	3 (2.9)	7 (6.7)
Angiographic eTICI scores^*^, n (%)												
2b50	0	0	0	0	38 (30.2)	33 (25.6)	17 (10.4)	20 (12.5)	na	na	6 (5.8)	2 (1.9)
2b67	0	0	0	0	49 (38.9)	44 (34.1)	82 (50)	69 (43.1)	na	na	14 (13.5)	8 (7.7)
2c	96 (36.0)	90 (33.7)	101 (37.5)	102 (37.6)	28 (22.2)	38 (29.5)	56 (34.2)	51 (31.9)	na	na	12 (11.5)	16 (15.4)
3	168 (62.9)	175 (65.5)	167 (62.1)	167 (61.6)	11 (8.7)	12 (9.3)	9 (5.5)	20 (12.5)	na	na	72 (69.2)	78 (75)
Angiographic eTICI scores subgroup, n (%)												
2b50/67	0	0	0	0	87 (69.0)	77 (59.9)	99 (60.4)	89 (55.6)	34 (56)	31 (60)	20 (19.3)	10 (9.6)
2c/3	267/267	267/267	269/269	271/271	39 (31.0)	50 (38.8)	65 (39.6)	71 (44.4)	27 (44)	21 (40)	84 (80.7)	94 (90.4)
Time from stroke onsite to randomization, h, median (IQR)	8.7 (5.2–13.0)	8.7 (5.3–13.6)	8.3 (5.1–12.6)	8.2 (5.4–13.5)	9.8 (6.7–14.0)	10.9 (7.0–15.5)	7.2 (4.4–11.7)	7.0 (5.0–11.5)	5.1 (3.5–11.2)	5.8 (4.0–10.2)	5.8 (4.0–8.4)	7.0 (3.8–9.4)

ASPECTS indicates Alberta Stroke Program Early CT [Computed Tomography] Score; BA, basilar artery; CHOICE, Intraarterial Alteplase Versus Placebo After Mechanical Thrombectomy; eTICI, expanded Thrombolysis in Cerebral Infarction; ICA, internal carotid artery; IQR, interquartile range; IVT, intravenous thrombolysis; M1, first segment of the middle cerebral artery; M2, second segment of the middle cerebral artery; NIHSS, National Institutes of Health Stroke Scale; PCA, posterior cerebral artery; and VA, vertebral artery.

* 2b50: reperfusion of 50% to66% of the territory; 2b67: reperfusion of 67% to89% of the territory; 2c: extensive reperfusion of 90% to99% of the territory; 3: complete or full reperfusion (100% reperfusion); 2b50/67: reperfusion of 50% to89% of the territory; 2c/3: extensive reperfusion of 90% to99% of the territory or 100% reperfusion.

^†^ The CHOICE study used intra–arterial infusion of placebo in the control group, whereas the other 5 studies did not administer any intra–arterial treatment in their control groups.

### Summary of Primary Efficacy and Safety Outcomes

All 6 studies used 90‐day mRS score 0–1 as the primary efficacy outcome. POST‐UK (adjusted RR: 1.13 [95% CI: 0.94–1.36]), POST‐TNK (adjusted RR: 1.15 [95% CI: 0.97–1.36]), and ATTENTION (adjusted RR: 1.36 95% CI: 0.92–2.02) reported neutral results, suggesting that patients with successful EVT may not benefit from adjunctive IAT. In contrast, ANGEL‐TNK (adjusted RR: 1.44 [95% CI: 1.06–1.95]), PEARL (adjusted RR: 1.45, 95% CI: 1.08–1.96), and CHOICE (adjusted risk difference [RD]: 18.4[95% CI: 0.3–36.4]) demonstrated that IAT after successful EVT could significantly improve functional outcomes. Regarding primary safety outcomes, all 6 RCTs indicated that IAT did not increase the risk of sICH or 90‐day mortality (Table 2).

**Table 2:** Summary of Primary Efficacy and Safety Outcomes

Study	Treatment	Control	Effect measure	Effect estimates	*P* value
Primary efficacy outcome: mRS score 0–1 at 90 d, n (%)					
POST‐UK	120/266 (45.1)	107/266 (40.2)	adjusted RR	1.13 (0.94–1.36)	0.19
POST‐TNK	132/269 (49.1)	119/270 (44.1)	adjusted RR	1.15 (0.97–1.36)	0.11
ANGEL‐TNK	51/126 (40.5)	34/129 (26.4)	adjusted RR	1.44 (1.06–1.95)	0.02
PEARL	73/164 (44.8)	48/160 (30.2)	adjusted RR	1.45 (1.08–1.96)	0.01
CHOICE	36/61 (59.0)	21/52 (40.4)	adjusted risk difference	18.4 (0.3–36.4)	0.047
ATTENTION‐IA	36/104 (34.6)	27/104 (26.0)	adjusted RR	1.36 (0.92–2.02)	0.12
Primary safety outcome: sICH^*^, n (%)					
POST‐UK	11/266 (4.1)	11/266 (4.1)	adjusted RR	1.05 (0.45–2.44)	0.91
POST‐TNK	17/268 (6.3)	12/271 (4.4)	adjusted RR	1.43 (0.68–2.99)	0.35
ANGEL‐TNK	7/126 (5.6)	8/129 (6.2)	adjusted RR	0.95 (0.36–2.53)	0.92
PEARL	7/164 (4.3)	8/160 (5.0)	adjusted RR	0.85 (0.43–1.69)	0.67
CHOICE	0	2/52 (3.8)	absolute risk difference	−3.8 (−13.2–2.5)	na
ATTENTION‐IA	8/104 (8.3)	3/104 (3.1)	adjusted RR	3.09 (0.78–12.20)	na
Primary safety outcome: mortality at 90 d, n (%)					
POST‐UK	49/266 (18.4)	46/266 (17.3)	adjusted hazard ratio	1.06 (0.71–1.59)	0.77
POST‐TNK	43/269 (16.0)	52/270 (19.3)	adjusted hazard ratio	0.75 (0.50–1.13)	0.16
ANGEL‐TNK	27/126 (21.4)	28/129 (21.7)	absolute RR	0.99 (0.62–1.58)	0.39
PEARL	28/164 (17.1)	18/160 (11.3)	adjusted hazard ratio	1.60 (0.88–2.89)	0.12
CHOICE	5/61 (8.2)	8/52 (15.4)	absolute risk difference	−7.2 (−19.2–4.8)	na
ATTENTION‐IA	29/104 (27.9)	28/104 (26.9)	adjusted RR	1.13 (0.73–1.74)	na

ANGEL‐TNK indicates Intra‐Arterial Recombinant Human TNK Tissue‐Type Plasminogen Activator Thrombolysis for Acute Large Vascular Occlusion After Successful Mechanical Thrombectomy Recanalization; ATTENTION‐IA, Intra‐Arterial Tenecteplase After Successful Endovascular Recanalisation in Patients With Acute Posterior Circulation Arterial Occlusion; CHOICE, Intraarterial Alteplase Versus Placebo After Mechanical Thrombectomy; mRS, modified Rankin Scale; PEARL, Intra‐Arterial Alteplase for Acute Ischaemic Stroke After Mechanical Thrombectomy; POST‐TNK, Adjunctive Intra‐Aarterial Tenecteplase Following Near‐Complete to Complete Reperfusion for Large Vessel Occlusion Stroke; POST‐UK, Adjunctive Intra‐Arterial Urokinase After Near‐Complete to Complete Reperfusion for Acute Ischemic Stroke; RR, risk ratio; and sICH, symptomatic intracranial hemorrhage.

### Summary of Subgroup Analysis of Primary Efficacy Outcome

The subgroup analysis based on eTICI scores (2b50/67 versus 2c/3) revealed conflicting results between studies. In CHOICE, the 2c/3 subgroup had improved functional outcomes (absolute RD: 32.3 [95% CI: 5.3–59.3]) whereas 2b50/67 patients did not (absolute RD: 8.1[95% CI: −16.1 to 32.2]). However, in ANGEL‐TNK the opposite trend was observed, with better outcome found only in the 2b50/67 subgroup (adjusted RR: 2.08 [95% CI: 1.35–3.20]) and not in the 2c/3 group (Table S2). Subgroup analysis of prior IVT was more consistent across the studies and indicated that patients without prior IVT benefited more from IAT than those who received prior IVT. For patients not receiving IVT in CHOICE, the absolute RD was 27.7 (95% CI: 2.5–53) and in PEARL, the adjusted RR was 1.91 (95% CI: 1.02–3.57) (Table S3).

Further subgroup analyses based on stroke etiology, admission National Institutes of Health Stroke Scale score, time from stroke onset to randomization, age, and sex were summarized in Tables S4–S8. POST‐UK and POST‐TNK had improved outcome in the subgroup with cardioembolism, with adjusted RRs of 1.49 (95% CI: 1.08–2.06) and 1.32 (95% CI: 1.02–1.71), respectively. Additionally, patients with lower admission National Institutes of Health Stroke Scale scores had better functional outcomes, with adjusted RRs of 1.64 (95% CI: 1.02–2.62) in ATTENTION, 1.48 (95% CI: 1.06–2.07) in ANGEL‐TNK, and 1.59 (95% CI: 1.20–2.10) in PEARL. Younger patients also had better functional recovery, with an adjusted RR of 1.42 (95% CI: 1.02–1.99) in ANGEL‐TNK and 1.48 (95% CI: 1.05–2.08) in PEARL.

### Meta‐Analysis of Primary Efficacy and Safety Outcomes

**Figure 2:**
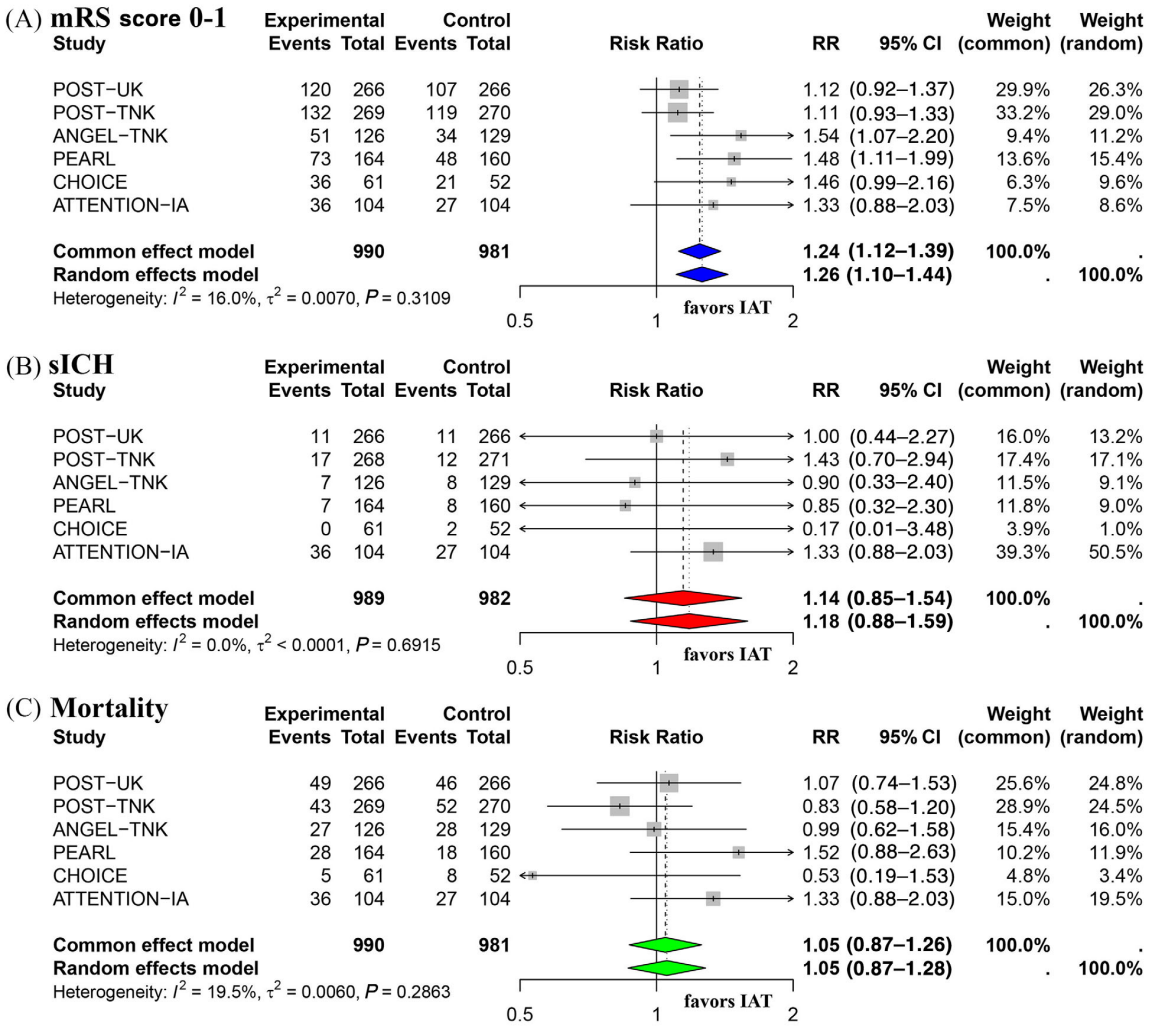
**Meta‐analysis of primary efficacy and safety outcomes**. **A**, Meta‐analysis of primary efficacy outcome: mRS score 0–1 at 90 days. **B**, Meta‐analysis of primary safety outcome: sICH. **C**, Meta‐analysis of primary safety outcome: mortality at 90 days. ANGEL‐TNK indicates Intra‐Arterial Recombinant Human TNK Tissue‐Type Plasminogen Activator Thrombolysis for Acute Large Vascular Occlusion After Successful Mechanical Thrombectomy Recanalization; ATTENTION‐IA, Intra‐Arterial Tenecteplase After Successful Endovascular Recanalisation in Patients With Acute Posterior Circulation Arterial Occlusion; IAT, intra‐arterial thrombolysis; mRS, modified Rankin Scale; PEARL, Intra‐Arterial Alteplase for Acute Ischaemic Stroke After Mechanical Thrombectomy; POST‐TNK, Adjunctive Intra‐Aarterial Tenecteplase Following Near‐Complete to Complete Reperfusion for Large Vessel Occlusion Stroke; POST‐UK, Adjunctive Intra‐Arterial Urokinase After Near‐Complete to Complete Reperfusion for Acute Ischemic Stroke; RR, risk ratio; and sICH, symptomatic intracranial hemorrhage.

A total of 990 patients were randomized to receive IAT following successful EVT, and 981 patients were assigned to the control group without further IAT treatment. Figure 2A shows the meta‐analysis results of the primary efficacy outcome (mRS score 0–1 at 90 days). Given the low heterogeneity (I^2^ = 16.0%, *P* = 0.31), a common‐effect model was applied, yielding a pooled RR of 1.24 (95% CI: 1.12–1.39), indicating that IAT treatment improved the rate of disability‐free survival at 90 days.

Among the 6 included RCTs, only CHOICE reported RD (18.4 [95% CI: 0.3–36.4]), whereas the others used RR. When we calculated RR (1.46 [95% CI: 0.99–2.16]) for CHOICE, its primary efficacy results shifted from positive to neutral. To address this discrepancy, we conducted a leave‐one‐out sensitivity analysis, systematically excluding each individual study one at a time to assess its impact on the overall pooled estimate. The results confirmed the stability of the pooled estimate, as the pooled RR remained within a narrow range (1.21–1.31) with minimal heterogeneity variation (I^2^: 3.1%–22.5%) (Figure S2).

Figure 2B,C present the meta‐analysis of primary safety outcomes. The common‐effect model demonstrated that IAT did not increase the risk of sICH (pooled RR: 1.14 [95% CI: 0.85–1.54]), with no observed heterogeneity (I^2^ = 0%, *P* = 0.70). Similarly, IAT did not increase 90‐day mortality (pooled RR: 1.05 [95% CI: 0.87–1.26]) and low heterogeneity (I^2^ = 20.2%, *P* = 0.28).

### Subgroup Meta‐Analysis of eTICI for Primary Efficacy Outcomes

**Figure 3:**
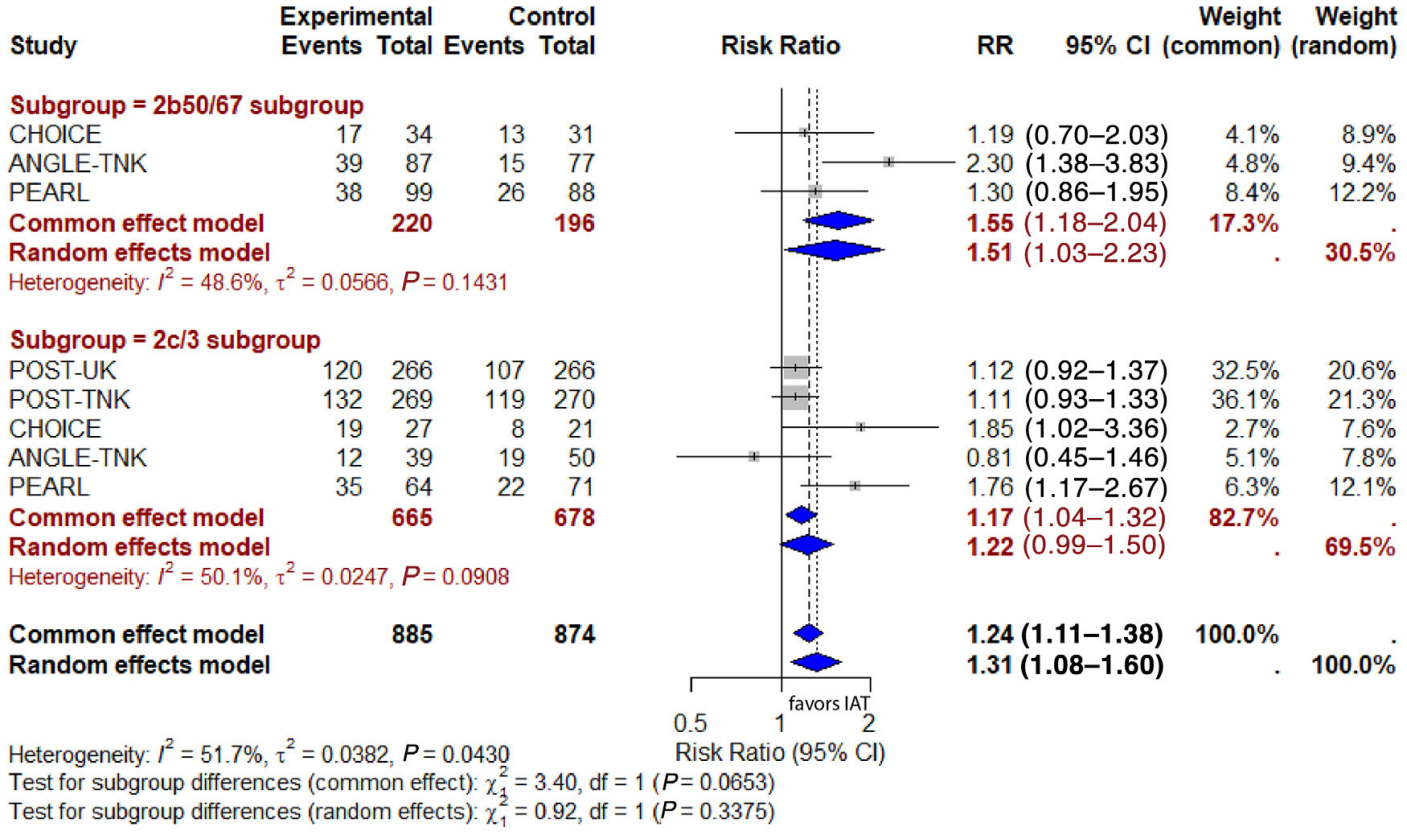
**Subgroup meta‐analysis of eTICI for primary efficacy outcomes**. ANGEL‐TNK indicates Intra‐Arterial Recombinant Human TNK Tissue‐Type Plasminogen Activator Thrombolysis for Acute Large Vascular Occlusion After Successful Mechanical Thrombectomy Recanalization; ATTENTION‐IAm Intra‐Arterial Tenecteplase After Successful Endovascular Recanalisation in Patients With Acute Posterior Circulation Arterial Occlusion; CHOICE, Intraarterial Alteplase Versus Placebo After Mechanical Thrombectomy; eTICI, expanded thrombolysis in cerebral infarction; IAT, intra‐arterial thrombolysis; POST‐TNK, Adjunctive Intra‐Aarterial Tenecteplase Following Near‐Complete to Complete Reperfusion for Large Vessel Occlusion Stroke; POST‐UK, Adjunctive Intra‐Arterial Urokinase After Near‐Complete to Complete Reperfusion for Acute Ischemic Stroke; and RR, risk ratio.

In the eTICI 2b50/67 subgroup, the pooled RR was 1.51 (95% CI: 1.03–2.23), suggesting IAT improved functional outcomes. A random‐effects model was applied due to moderate heterogeneity (I^2^ = 48.6%, *P* = 0.14). Conversely, in the eTICI 2c/3 subgroup, the pooled RR was 1.22 (95% CI: 0.99–1.50), with heterogeneity measured at I^2^ = 50.1%, *P* = 0.09. The subgroup difference test (random‐effects model) yielded χ^2^ = 0.92, *P* = 0.34, indicating no significant difference between the subgroups (Figure 3).

### Subgroup Meta‐Analysis of Prior IVT for Primary Efficacy Outcomes

**Figure 4:**
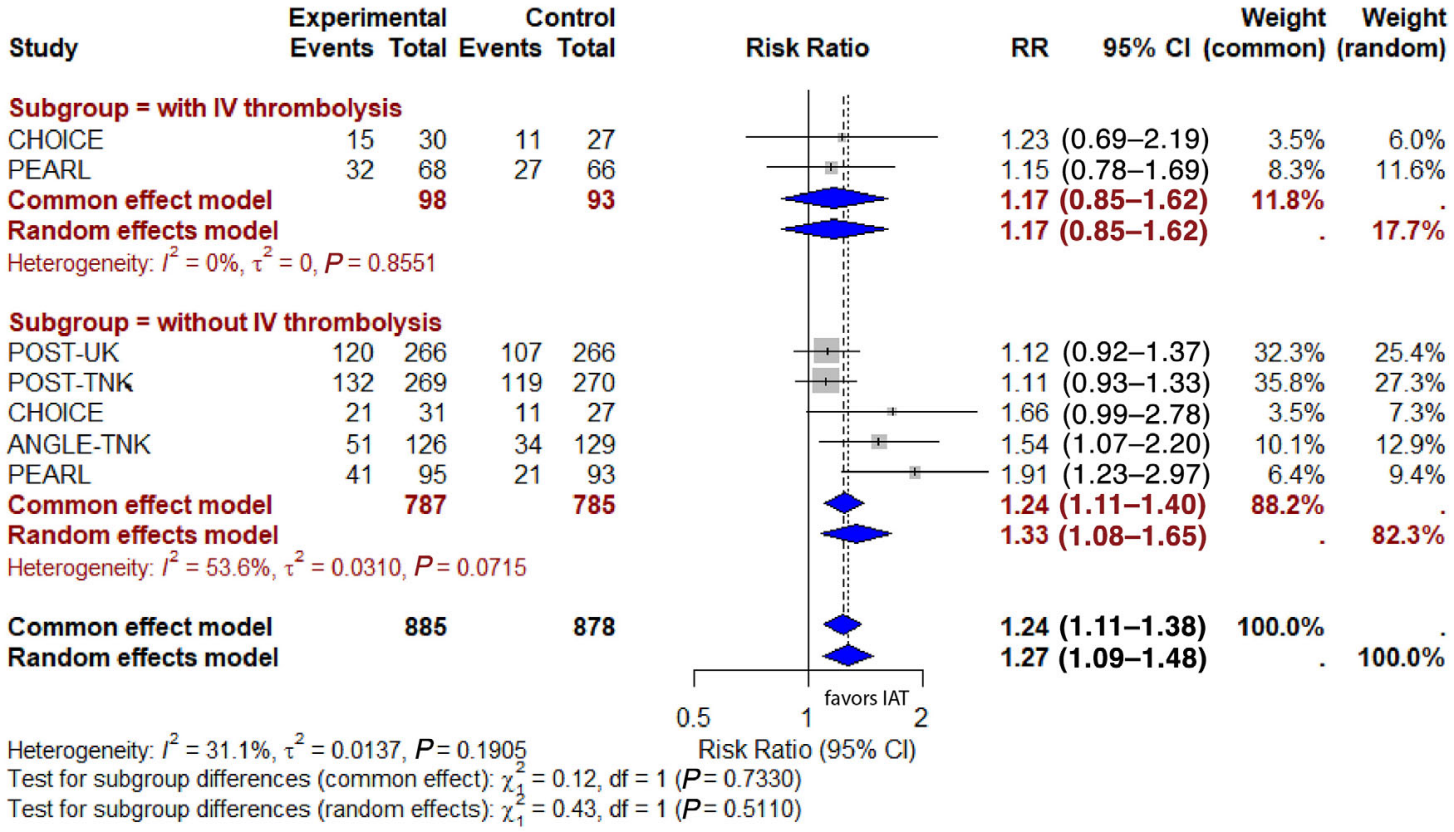
**Subgroup meta‐analysis of prior IVT for primary efficacy outcomes**. ANGEL‐TNK indicates Intra‐Arterial Recombinant Human TNK Tissue‐Type Plasminogen Activator Thrombolysis for Acute Large Vascular Occlusion After Successful Mechanical Thrombectomy Recanalization; ATTENTION‐IA, Intra‐Arterial Tenecteplase After Successful Endovascular Recanalisation in Patients With Acute Posterior Circulation Arterial Occlusion; CHOICE, Intraarterial Alteplase Versus Placebo After Mechanical Thrombectomy; IAT, intra‐arterial thrombolysis; IV, intravenous; IVT, intravenous thrombolysis; PEARL, Intra‐Arterial Alteplase for Acute Ischaemic Stroke After Mechanical Thrombectomy; POST‐TNK, Adjunctive Intra‐Aarterial Tenecteplase Following Near‐Complete to Complete Reperfusion for Large Vessel Occlusion Stroke; POST‐UK, Adjunctive Intra‐Arterial Urokinase After Near‐Complete to Complete Reperfusion for Acute Ischemic Stroke; and RR, risk ratio.

In the subgroup who did not receive IVT prior to EVT, the pooled RR, estimated using the random‐effects model was 1.33 (95% CI: 1.08–1.65), suggesting that patients without prior IVT did benefit from IAT treatment following successful EVT. The heterogeneity was moderate (I^2^ = 53.6%, *P* = 0.07). The prior IVT subgroup was based on a limited sample size from only two of the studies (CHOICE and ANGEL‐TNK). The pooled RR was 1.17 (95% CI: 0.85–1.62), with no heterogeneity (I^2^ = 0, *P* = 0.85). The subgroup difference test (random effect) yielded χ^2^ = 0.43, *P* = 0.51, indicating no significant difference between the subgroups (Figure 4).

## Discussion

This meta‐analysis showed that IAT following successful EVT significantly improved the rate of disability‐free survival without raising the risk of sICH or death. Subgroup meta‐analysis revealed a trend toward greater therapeutic benefits from IAT in the eTICI 2b50/67 subgroup compared with the eTICI 2c/3 subgroup, and greater benefits in patients without prior IVT.

One challenge of EVT treatment is to bridge the asymmetry between the high rate of “successful” large vessel recanalization with the much lower rate of good functional outcomes.^17^, ^18^ Impaired microcirculatory reperfusion has been reported as a key contributing factor for “futile” recanalization.^19^ Rubiera et al^20^ have reported that 52.9% of patients in their study exhibited varying degrees of persistent perfusion defects on post‐EVT computed tomography perfusion imaging, despite achieving successful recanalization. Moreover, patients with post‐EVT hypoperfusion were reported to experience larger final infarct volume and poorer functional recovery.^21^, ^22^ Infarct adjunctive IAT after EVT has been proposed as a strategy to dissolve residual thrombi or emboli that migrate into microcirculation, thereby enhancing microvascular perfusion and improving clinical outcomes.

In this study, the meta‐analysis of the eTICI subgroup found that patients in the 2b50/67 subgroup tend to benefit more from IAT compared with those in the 2c/3 subgroup. Patients with eTICI 2b50/67 achieve only partial reperfusion of the affected territory, whereas 2c/3 patients attain nearly complete reperfusion.^10^ Consequently, residual thrombus or distal thrombus migration are more likely to exist in 2b50/67 patients, potentially making them more responsive to IAT. The CHOICE trial was an outlier among the 6 RCTs, paradoxically reporting an improved outcome with IAT in the 2c/3 subgroup but not in the 2b50/67 subgroup. The CHOICE trial was prematurely terminated due to the COVID‐19 pandemic, with the final sample size reaching only 60% of the planned enrollment.^6^

The meta‐analysis on IVT subgroups suggested that patients who do not receive prior IVT are likely to benefit from IAT following successful EVT. Currently, IVT remains a cornerstone of acute ischemic stroke treatment,^23^ but the treatment is predominantly limited to 4.5 h from stroke onset. At this point, the generalizability of the findings may be limited to endovascular capable centers only, where direct EVT and postprocedural IAT are an afforded approach. For primary stroke centers, the current findings do not justify limiting IVT prior to transfer for EVT based on the current meta‐analysis.

However, the prior IVT subgroup is based on a limited sample size with 98 patients in the IAT group and 93 in the control group. Given the small sample, these findings should be interpreted with caution. Real‐world data indicate that 63.2%–75.5% of EVT‐treated patients receive prior IVT.^24^ Further RCTs are needed to confirm whether IAT offers meaningful benefits for patients who have undergone prior IVT.

Notably, none of the 6 RCTs selected patients with direct evidence microcirculation dysfunction – so‐called “no‐reflow phenomenon” – although it is considered as the target of the postthrombectomy thrombolysis.^21^, ^25^ The therapeutic effect of IAT may be further improved if patient selection in future studies is refined by identifying with microcirculation dysfunction after successful EVT. This might be achieved using digital subtraction angiography “perfusion” and flat‐panel computed tomography perfusion.^26^

There are 2 other meta‐analyses also focusing on the same topic. Yang et al^27^ included only 4 RCTs, excluding the PEARL and ANGEL‐TNK trials, which have published only their protocols and presented results at international conference. Guo et al^28^ included 7 RCTs by adding the Optimal Dosage of Adjunctive Intra‐Arterial Tenecteplase Following Successful Endovascular Thrombectomy in Patients With Large Vessel Occlusion Acute Ischemic Stroke (DATE) trial, whose both protocol and results have not yet been peer‐reviewed, thereby introducing more bias. Our meta‐analysis included six RCTs without the DATE trial, in an effort to strike a balance between increasing the amount of available data and minimizing potential bias. More important, our study placed greater emphasis on subgroup analyses of recanalization status and prior IVT treatment. These 2 subgroup analyses are mechanistically more relevant, reflecting the treatment target of IAT. They may help clinicians identify which patients are most likely to benefit from adjunctive IAT.

This meta‐analysis has several limitations. First, among the 6 included RCTs, the CHOICE trial reported the risk difference of the intervention group and control group, wjereas the other trials reported the RR. When we calculated the RR from the CHOICE data for the meta‐analysis, its primary efficacy outcome shifted from positive to neutral. Risk difference reflects the absolute difference between the intervention and control groups, such as preventing 2 events per 100 patients treated. In contrast, the RR represents the relative effect, such as a 25% risk reduction in the intervention group. In addition, risk difference is based on a normal distribution, whereas RR relies on logarithmic transformation. When the sample size is small, the CI for RR is more likely to cross 1, making the result statistically nonsignificant. However, our sensitivity analysis showed that excluding CHOICE did not affect the overall conclusion. Second, the ATTENTION‐AI trial did not report the exact number and proportion of patients in each subgroup who achieved the primary efficacy outcome, preventing its inclusion in our subgroup meta‐analysis. Third, the definitions of sICH varied across studies. Although each RCT and our meta‐analysis found that IAT did not increase the risk of sICH, this variation may affect result comparability. Similarly, differences in the type and dosage of thrombolytic agents used across studies may also affect the comparability of findings. Fourth, all 6 RCTs used mRS score 0–1 at 90 days as the primary efficacy outcome. However, mRS score 0–2 is a more commonly used measure to define good clinical outcomes. This may raise questions regarding the comparability or generalizability of the results. Finally, the ANGEL‐TNK and PEARL studies presented their results at International Stroke Conference 2025 without having been peer reviewed. Therefore, we conducted a leave‐one‐out sensitivity analysis and found that excluding either of the 2 studies did not change the overall results of the meta‐analysis. Specifically, the pooled RR for the primary efficiency outcome was 1.21 (95% CI: 1.09–1.36) when omitting ANGEL‐TNK, and 1.21 (95% CI: 1.08–1.35) when omitting PEARL (Figure S2). In addition, the formally published protocols of both studies help support their methodological quality and transparency.^15^, ^16^ Nevertheless, the results of these 2 studies should still be interpreted with caution.

In conclusion, IAT following successful EVT can improve 90‐day functional outcomes of patients with stroke, without increasing the risk of sICH or 90‐day mortality. The ideal target population currently for IAT might be those without complete reperfusion from EVT and in patients with no prior thrombolysis. Further RCTs are required to better define its role.

## Supporting information




**Appendix 1**: Detailed search strategy
**Table S1**: Summary of RCTs Protocols
**Table S2**: Summary of Subgroup analysis: eTICI
**Table S3**: Summary of Subgroup analysis: Prior Intravenous thrombolysis
**Table S4**: Summary of Subgroup analysis: Stroke Etiology
**Table S5**: Summary of Subgroup analysis: Admission NIHSS
**Table S6**: Summary of Subgroup analysis: Time from Stroke Onset to Randomization
**Table S7**: Summary of Subgroup analysis: Age
**Table S8**: Summary of Subgroup analysis: sex
**Figure S1**: Quality Assessment: Risk of Bias 2.0
**Figure S2**: Sensitivity analysis: Leave‐One‐Out Analysis of Primary Efficiency Outcomes
