# Supplementary material for: Intra‐Arterial Thrombolysis After Successful Thrombectomy: A Systematic Review and Meta‐Analysis of Randomized Controlled Trials
Source: Stroke Vasc Interv Neurol. 2025 Jul 31;5(5):e001847. doi: 10.1161/SVIN.125.001847 (PMC12697620; doi:10.1161/SVIN.125.001847)

SUPPLEMENTAL MATERIALS

Intra-Arterial Thrombolysis After Successful Thrombectomy: A Systematic Review and Meta-Analysis of Randomized Controlled Trials

Short title: IAT after EVT, a meta-analysis of RCTs

Tianqi Xu, MD[1,2*], Chushuang Chen, PhD[1,2], Vignan Yogendrakumar, PhD[5], Dennis J Cordato, PhD[1,2,3], Christopher Blair, PhD[1,2,3], Timmy Pham, Master[1,2,3], Andrew K Cheung, FRANZCR[1,2,4], Nathan W Manning, FRANZCR[1,2,4], Mark W Parsons, PhD[1,2,3], Longting Lin, PhD[1,2#]

1. South Western Sydney Clinical School, UNSW, Liverpool, NSW, 2170, Australia

2. Ingham Institute for Applied Medical Research, Liverpool, NSW, 2170, Australia

3. Department of Neurology, Liverpool Hospital, Sydney, NSW, 2170, Australia

4. Department of Neurointerventional Radiology, Liverpool Hospital, NSW, 2170, Australia

5. Division of Neurology, Department of Medicine, The Ottawa Hospital, Ottawa Hospital Research Institute, University of Ottawa, Ottawa, ON, Canada.

# Corresponding Author:

Longting Lin, PhD, Email: longting.lin@unsw.edu.au, South Western Sydney Clinical School, UNSW, Liverpool, NSW, 2170, Australia ＆ Ingham Institute for Applied Medical Research, Liverpool, NSW, 2170, Australia

Appendix 1: Detailed search strategy

Search strategy in PubMed:

("stroke"[MeSH] OR "brain ischemia"[MeSH] OR stroke[tiab] OR "brain ischemia"[tiab] OR " cerebrovascular accident"[tiab])

AND

(("Intra-Arterial"[MeSH] OR "intra-arterial"[tiab] OR "intraarterial"[tiab])

AND

("Thrombolytic Therapy"[MeSH] OR "tissue plasminogen activator"[MeSH] OR alteplase[tiab] OR urokinase[tiab] OR "tenecteplase"[tiab] OR "thrombolysis"[tiab]))

AND

("Thrombectomy"[MeSH] OR "mechanical thrombectomy"[tiab] OR "endovascular thrombectomy"[tiab])

AND

("Randomized Controlled Trial"[Publication Type] OR "randomized controlled trial"[tiab] OR "randomised controlled trial"[tiab] OR "randomized clinical trial"[tiab] OR "randomised clinical trial"[tiab] OR "randomized trial"[tiab] OR "randomised trial"[tiab] OR "RCT"[tiab])

Search strategy in EMBASE:

("cerebrovascular disease" OR "cerebrovascular accident" OR "stroke" OR "strokes" OR "large vessel occlusion" OR "brain ischemia" OR "brain infarction" OR "cerebral infarction" OR "cerebral ischemia" OR "middle cerebral artery" OR "MCA" OR "anterior circulation" OR "posterior circulation" OR thrombus OR occlusion).ab,ti.

AND

(("intraarterial" OR "intra-arterial" OR "intra-arterial therapy").ab,ti.

AND

("thrombolysis" OR "thrombolytic therapy" OR "tissue plasminogen activator"

OR alteplase OR urokinase OR tenecteplase).ab,ti.)

AND

("thrombectomy" OR "mechanical thrombectomy" OR "endovascular thrombectomy").ab,ti.

AND

("randomized controlled trial" OR "randomized trial" OR "randomised controlled trial" OR "randomised trial" OR "randomized clinical trial" OR "randomised clinical trial" OR "RCT").ab,ti.

Table S1: Summary of RCTs Protocols

Study	Design	Sample Size	Inclusion criteria	Intervention	Control	Primary efficacy outcome	Primary safety outcomes
POST-UK	Randomized, open-label, blinded-endpoint trial in 35 stroke center across China	535	1. LVO in the anterior circulation.<br>2. Achieve eTICI 2c-3.<br>3. Without IV treatment	intra-arterial infusion of urokinase 100 000 IU for 10 to 15 minutes.	without further intra-arterial thrombolysis.	mRS 0-1 at 90 days	1. Mortality at 90 days.<br>2. sICH within 48 hours.
POST-TNK	Randomized, open-label, blinded–endpoint trial in 34 stroke center across China	540	1. LVO in the anterior circulation.<br>2. Achieve eTICI 2c-3.<br>3. Without IV treatment	intra-arterial infusion of TNK 0.0625 mg/kg (maximum 6.25mg) for 10 to 15minutes.	without further intra-arterial thrombolysis.	mRS 0-1 at 90 days	1. Mortality at 90 days.<br>2. sICH within 48 hours.
CHOICE	Phase 2b randomized, double-blind, placebo-controlled trial in 7 center in Spain	121	1. LVO in anterior circulation.<br>2. Achieve mTICI 2b-3.	intra-arterial infusion of alteplase (0.225 mg/kg; maximum 22.5 mg) for 15 to 30 minutes.	intra-arterial infusion of placebo	mRS 0-1 at 90 days	1. Mortality at 90 days.<br>2. sICH within 24 hours.
ATTENTION-IA	Randomized, open-label, blinded–endpoint trial in 31 stroke center across China	208	1. LVO in posterior circulation.<br>2. Achieve eTICI 2b50-3.	Intra-arterial infusion of TNK (0.0625 mg/kg, maximum 6.25 mg) over 15 seconds.	without further intra-arterial thrombolysis.	mRS 0-1 at 90 days	1. Mortality at 90 days.<br>2. sICH within 36 hours.
ANGEL-TNK	Randomized, open-label, blinded–endpoint trial in 19 stroke center across China	255	1. LVO in the anterior circulation.<br>2. Achieve eTICI 2b50-3.<br>3. Without IV treatment	intra-arterial infusion of TNK 0.125 mg/kg (maximum12.5 mg) for more than 15 minutes.	without further intra-arterial thrombolysis.	mRS 0-1 at 90 days	1. Mortality at 90 days.<br>2. sICH within 48 hours.
PEARL	Randomized, open-label, blinded–endpoint trial in 28 stroke center across China	324	1. LVO in the anterior circulation.<br>2. Achieve eTICI 2b50-3.	intra-arterial infusion of alteplase 0.225 mg/kg (maximum 20 mg) for 15 minutes.	without further intra-arterial thrombolysis.	mRS 0-1 at 90 days	1. Mortality at 90 days.<br>2. sICH within 36 hours.

Figure S1: Quality Assessment: Risk of Bias 2.0

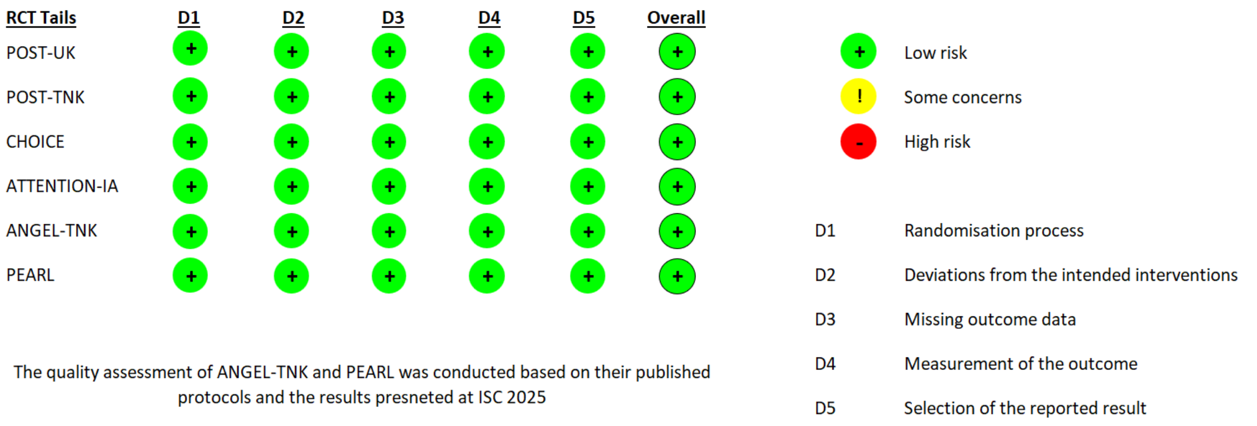

Table S2: Summary of Subgroup analysis: eTICI

RCT trails	eTICI	Treatment	Control	Effect Measure	Effect Estimate	P value for interaction
POST-UK						na
	0	0	0	na	na	
	2c/3	120/266 (45.1)	107/266 (40.2)	adjusted risk ratio	1.13 (0.94-1.36)	
POST-TNK						na
	0	0	0	na	na	
	2c/3	132/269 (49.1)	119/270 (44.1)	adjusted risk ratio	1.15 (0.97-1.36)	
CHOICE						0.468
	2b50/67	17/34 (50.0)	13/31 (41.9)	absolute risk difference	8.1 (-16.1-32.2)	
	2c/3	19/27 (70.4)	8/21 (38.1)	absolute risk difference	32.3 (5.3-59.3)	
ATTENTION-IA						na
	2b50/67	na	na	adjusted risk ratio	0.92 (0.41-2.08)	
	2c/3	na	na	adjusted risk ratio	1.89 (0.96-3.70)	
ANGEL-TNK						0.09
	2b50/67	39/87 (44.8)	15/77 (19.5)	adjusted risk ratio	2.08 (1.35-3.20)	
	2c/3	12/39 (30.8)	19/50 (38.0)	adjusted risk ratio	0.84 (0.29-2.42)	
PEARL						0.42
	2b50/67	38/99 (38.4)	26/88 (29.6)	adjusted risk ratio	1.3 (0.88-1.92)	
	2c/3	35/64 (54.7)	22/71 (31.0)	adjusted risk ratio	1.6 (0.95-2.68)	

Abbreviations: eTICI: Expanded Thrombolysis in Cerebral Infarction

Table S3: Summary of Subgroup analysis: Prior Intravenous thrombolysis

RCT trails	Intravenous thrombolysis	Treatment	Control	Effect Measure	Effect Estimate	P value for interaction
POST-UK						na
	No	120/266 (45.1)	107/266 (40.2)	adjusted risk ratio	1.13 (0.94-1.36)	
	Yes	0	0	na	na	na
POST-TNK						
	No	132/269 (49.1)	119/270 (44.1)	adjusted risk ratio	1.15 (0.97-1.36)	
	Yes	0	0	na	na	
CHOICE						0.367
	No	21/31 (67.7)	10/25 (40.0)	absolute risk difference	27.7 (2.5-53)	
	Yes	15/30 (50.0)	11/27 (40.7)	absolute risk difference	9.3 (-16.5-35)	
ATTENTION-IA						na
	No	na	na	adjusted risk ratio	1.34 (0.86-2.09)	
	Yes	na	na	adjusted risk ratio	1.53 (0.63-3.68)	
ANGEL-TNK						na
	No	51/126 (40.5)	34/129 (26.4)	adjusted risk ratio	1.44 (1.06-1.95)	
	Yes	0	0	na	na	
PEARL						0.13
	No	41/95 (43.2)	21/93 (22.6)	adjusted risk ratio	1.91 (1.02-3.57)	
	Yes	32/68 (47.1)	27/66 (40.9)	adjusted risk ratio	1.15 (0.91-1.46)	

Table S4: Summary of Subgroup analysis: Stroke Etiology

RCT trails	Stroke etiology	Treatment	Control	Effect Measure	Effect Estimate	P value for interaction
POST-UK						0.1
	Large artery atherosclerosis	59/135 (43.7)	60/130 (46.2)	adjusted risk ratio	0.95 (0.74-1.23)	
	Cardioembolism	48/103 (46.6)	34/106 (32.1)	adjusted risk ratio	1.49 (1.08-2.06)	
	Other and unknown	13/28 (46.4)	13/30 (43.3)	adjusted risk ratio	1.25 (0.70-2.24)	
POST-TNK						0.58
	Large artery atherosclerosis	58/111 (52.3)	52/106 (49.1)	adjusted risk ratio	1.04 (0.81-1.33)	
	Cardioembolism	65/135 (48.2)	53/137 (38.7)	adjusted risk ratio	1.32 (1.02-1.71)	
	Other and unknown	9/23 (39.1)	14/27 (51.9)	adjusted risk ratio	0.88 (0.46-1.68)	
ATTENTION-IA						na
	Large artery atherosclerosis	na	na	adjusted risk ratio	1.66 (0.99-2.78)	
	Cardioembolism	na	na	adjusted risk ratio	0.82 (0.35-1.92)	
	Other and unknown	na	na	adjusted risk ratio	1.79 (0.64-4.96)	
ANGEL-TNK						0.95
	Large artery atherosclerosis	10/30 (33.3)	9/37 (24.3)	adjusted risk ratio	1.38 (0.67-2.82)	
	Cardioembolism	38/89 (42.7)	23/83 (27.7)	adjusted risk ratio	1.38 (0.97-1.98)	
	Other and unknown	na	na	na	na	

Table S5: Summary of Subgroup analysis: Admission NIHSS

RCT trails	Admission NIHSS	Treatment	Control	Effect Measure	Effect Estimate	P value for interaction
POST-UK						0.45
	≤15	81/144 (56.3)	77/145 (53.1)	adjusted risk ratio	1.08 (0.88-1.32)	
	>15	39/122 (32.0)	30/121 (24.8)	adjusted risk ratio	1.16 (0.79-1.72)	
POST-TNK						0.11
	≤15	79/138 (57.3)	85/149 (57.1)	adjusted risk ratio	1.03 (0.86-1.25)	
	>15	53/131 (40.5)	34/121 (28.1)	adjusted risk ratio	1.38 (0.99-1.92)	
ATTENTION-IA						0.16
	6-19	na	na	adjusted risk ratio	1.64 (1.02-2.62)	
	≥20	na	na	adjusted risk ratio	0.93 (0.45-1.94)	
ANGEL-TNK						0.79
	≤15	38/71 (53.3)	23/63 (36.5)	adjusted risk ratio	1.48 (1.06-2.07)	
	>15	13/55 (23.6)	11/66 (16.7)	adjusted risk ratio	1.14 (0.73-1.80)	
PEARL						0.53
	≤15	53/88 (60.2)	35/91 (38.5)	adjusted risk ratio	1.59 (1.20-2.10)	
	>15	20/75 (26.7)	13/68 (19.1)	adjusted risk ratio	1.29 (0.79-2.11)	

Abbreviations: NIHSS: National Institutes of Health Stroke Scale.

Table S6: Summary of Subgroup analysis: Time from Stroke Onset to Randomization

RCT trails	Time form stroke onset to randomization	Treatment	Control	Effect Measure	Effect Estimate	P value for interaction
POST-UK						0.51
	≤ 8.7 h	60/134 (44.8)	49/133 (36.8)	adjusted risk ratio	1.23 (0.93-1.63)	
	＞8.7 h	60/132 (45.5)	58/133 (43.6)	adjusted risk ratio	1.08 (0.84-1.39)	
POST-TNK						0.23
	≤ 8.3 h	70/134 (52.2)	63/135 (46.7)	adjusted risk ratio	1.28 (1.02-1.61)	
	＞8.3 h	62/135 (45.9)	56/135 (41.5)	adjusted risk ratio	1.02 (0.79-1.32)	
CHOICE						0.917
	≤ 7.3 h	23/38 (60.5)	13/31 (41.9)	difference	18.6 (-4.7-41.9)	
	＞7.3 h	13/23 (56.5)	8/21 (38.1)	difference	18.4 (-10.6-47.4)	
ATTENTION-IA						na
	＜ 6 h	na	na	adjusted risk ratio	1.06 (0.60-1.89)	
	≥ 6 h	na	na	adjusted risk ratio	1.63 (0.91-2.91)	
ANGEL-TNK						0.69
	4.5h-6h	9/15 (46.7)	5/17 (29.4)	adjusted risk ratio	1.62 (0.86-3.04)	
	6h-24h	38/97 (39.2)	27/100 (27.0)	adjusted risk ratio	1.30 (0.99-1.70)	

Table S7: Summary of Subgroup analysis: Age

RCT trails	Age	Treatment	Control	Effect Measure	Effect Estimate	P value for interaction
POST-UK						0.91
	≤69	68/135 (50.4)	69/147 (46.9)	adjusted risk ratio	1.11 (0.88-1.39)	
	＞69	52/131 (39.7)	38/119 (31.9)	adjusted risk ratio	1.15 (0.84-1.58)	
POST-TNK						0.92
	≤69	77/135 (57.0)	75/141 (53.2)	adjusted risk ratio	1.13 (0.93-1.39)	
	＞69	55/134 (41.0)	44/129 (34.1)	adjusted risk ratio	1.15 (0.86-1.53)	
ATTENTION-IA						na
	≤69	na	na	adjusted risk ratio	1.29 (0.81-2.05)	
	＞69	na	na	adjusted risk ratio	1.57 (0.77-3.21)	
ANGEL-TNK						0.92
	≤65	20/39 (51.3)	14/45 (31.1)	adjusted risk ratio	1.42 (1.02-1.99)	
	＞65	31/87 (35.6)	20/84 (23.8)	adjusted risk ratio	1.45 (0.83-2.54)	
PEARL						0.9
	≤65	45/72 (62.5)	29/69 (42.0)	adjusted risk ratio	1.48 (1.05-2.08)	
	＞65	28/91 (30.8)	19/90 (21.1)	adjusted risk ratio	1.40 (0.9-2.19)	

Table S8: Summary of Subgroup analysis: sex

RCT trails	Sex	Treatment	Control	Effect Measure	Effect Estimate	P value for interaction
POST-UK						0.06
	Female	49/105 (46.7)	38/118 (32.2)	adjusted risk ratio	1.47 (1.07-2.02)	
	male	71/161 (44.1)	69/148 (46.6)	adjusted risk ratio	0.97 (0.77-1.22)	
POST-TNK						0.14
	Female	61/115 (53.0)	42/105 (40.0)	adjusted risk ratio	1.31 (1.00-1.72)	
	male	71/154 (46.1)	77/165 (46.7)	adjusted risk ratio	1.02 (0.82-1.27)	
CHOICE						0.435
	Female	15/28 (53.6)	7/24 (29.2)	absolute risk difference	24.4 (-1.5-50.3)	
	male	21/33 (63.6)	14/28 (50.0)	absolute risk difference	13.6 (-11.1-38.4)	

Figure S2: Sensitivity analysis: Leave-One-Out Analysis of Primary Efficiency Outcomes

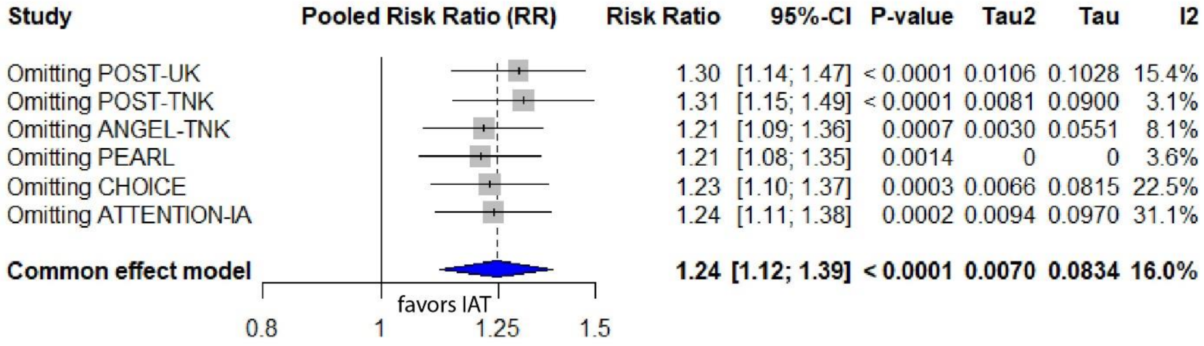

Supplement: Supplementary file 1 — Appendix 1: Detailed search strategy Table S1: Summary of RCTs Protocols Table S2: Summary of Subgroup analysis: eTICI Table S3: Summary of Subgroup analysis: Prior Intravenous thrombolysis Table S4: Summary of Subgroup analysis: Stroke Etiology Table S5: Summary of Subgroup analysis: Admission NIHSS Table S6: Summary of Subgroup analysis: Time from Stroke Onset to Randomization Table S7: Summary of Subgroup analysis: Age Table S8: Summary of Subgroup analysis: sex Figure S1: Quality Assessment: Risk of Bias 2.0 Figure S2: Sensitivity analysis: Leave‐One‐Out Analysis of Primary Efficiency Outcomes [file SVI2-5-e001847-s001.pdf]
